# Fast-Track Long Term Continuous Heart Monitoring in a Stroke Clinic: A Feasibility Study

**DOI:** 10.3389/fneur.2019.01400

**Published:** 2020-01-21

**Authors:** Ayesha Khan, Vida Abedi, Farhan Ishaq, Alireza Sadighi, Mohammad Adibuzzaman, Martin Matsumura, Neil Holland, Ramin Zand

**Affiliations:** ^1^Geisinger Neuroscience Institute, Geisinger Health System, Danville, PA, United States; ^2^Department of Molecular and Functional Genomics, Weis Center for Research, Geisinger Health System, Danville, PA, United States; ^3^Regenstrief Center for Healthcare Engineering, Purdue University, West Lafayette, IN, United States; ^4^Geisinger Health System, Pearsall Heart Hospital, Wilkes Barre, PA, United States

**Keywords:** paroxysmal atrial fibrillation, feasibility, stroke, cardiac monitoring, arrhythmia, transient ischemic attack (TIA), arrhythmias, atrial fibrillation.

## Abstract

**Background:** Paroxysmal atrial fibrillation (PAF) or flutter is prevalent among patients with cryptogenic stroke. The goal of this study was to investigate the feasibility of incorporating a fast-track, long term continuous heart monitoring (LTCM) program within a stroke clinic.

**Method:** We designed and implemented a fast-track LTCM program in our stroke clinics. The instrument that we used for the study was the ZioXT® device from IRhythm™ Technologies. To implement the program, all clinic support staff received training on the skin preparation and proper placement of the device. We prospectively followed every patient who had a request from one of our inpatient or outpatient stroke or neurology providers to receive LTCM. We recorded patients' demographics, the LTCM indication, as well as related quality measures including same-visit placement, wearing time, analyzable time, LTCM application to the preliminary finding time, as well as patients' out of pocket cost.

**Results:** Out of 501 patients included in the study, 467 (93.2%) patients (mean age 65.9 ± 13; men: 48%) received LTCM; and 92.5% of the patients had the diagnosis of stroke or TIA. 93.7% of patients received their LTCM during the same outpatient visit in the stroke clinic. The mean wearing time for LTCM was 12.1 days (out of 14 days). The average analyzable time among our patients was 95.0%. Eighteen (3.9%, 95%CI: 2.4–6.0) patients had at least one episode of PAF that was sustained for more than 30 s. The rate of PAF was 5.9% (95% CI: 3.5–9.2) among patients with the diagnosis of stroke. Out of 467 patients, 392 (84%) had an out-of-pocket cost of < $100.

**Conclusion:** It is feasible to implement a fast-track cardiac monitoring as part of a stroke clinic with proper training of stroke providers, clinic staff, and support from a cardiology team.

## Introduction

The number of adults with atrial fibrillation (AF) is estimated to be around 2.3 million in the United States (1). AF causes a 5-fold increase in the risk of ischemic stroke and accounts for approximately 15% of all strokes nationally (2). In one study, the prevalence of AF among patients with ischemic stroke was found to be 18%. Strokes linked to AF were also associated with increased morbidity and mortality compared to strokes of other etiologies (3, 4).

Screening for paroxysmal atrial fibrillation or flutter (PAF) after ischemic stroke or transient ischemic attack (TIA) should be considered for secondary stroke prevention. Apart from screening of PAF, cardiac monitoring can be utilized to diagnose cardiac arrhythmias that are associated with syncope. Several cardiac devices are available that are capable of remote assessment of ambulatory patients and intermittent or continuous recording. They can be used externally or as a subcutaneous implant (5, 6).

In many institutions, the heart monitoring process among patients with suspected embolic stroke, is managed by the cardiology department (7). In winter 2016, as part of our comprehensive stroke program, we began to incorporate the heart monitoring process in our stroke clinics to improve access, streamline and expedite post-stroke evaluation. Since we have been prospectively monitoring the workflow, the device placement procedure, and relevant heart monitoring findings. The goal of this study was to investigate the feasibility of incorporating a fast-track, long-term continuous heart monitoring (LTCM) program as part of a stroke clinic. We further evaluated the relevant LTCM findings.

## Methods

### Program Design and Implementation

For the purpose of this study, we designed and implemented a fast-track LTCM program in our stroke clinics in December 2016. The ZioXT® device from IRhythm™ Technologies was used (8). ZioXT is a single-lead, single-use, FDA-approved device that is worn externally. It provides uninterrupted ambulatory cardiac monitoring and can monitor heart rhythm for up to 14 days. The device can also be triggered when patients experience symptoms such as palpitations, dizziness, light-headedness, pre-syncope or shortness of breath. The device characteristics and placement protocol have previously been published (9, 10). At the end of 2-week monitoring period, patients send the device back to the company for analysis. The analysis is performed by a deep learning algorithm that can detect and classify arrhythmias. A certified cardiographic technician reviews and verifies the preliminary results and prepares a preliminary summary report. The preliminary reports are uploaded to a web portal where they could be accessed by patients' providers. Every preliminary report is reviewed and verified by one of our cardiologists before being uploaded in the electronic health records. The vascular neurology team examines the final interpretation of the results.

To implement the program, all stroke clinic nurses and support staff received training on skin preparation and proper placement of the device. The half-day training was done by our certified cardiology technicians from the Geisinger Heart Institute. After the initial training, our new recruited support staff were trained by previously-trained staff members in our stroke clinics. Each support staff had the opportunity to observe and be assisted in 10 LTCM applications before they apply the patch independently. The manufacturer representative arranged two 1 hour sessions to train the support staff regarding the ordering process, insurance coverage, access to the portal, device application process, and post application care. Stroke specialists also received training on the ordering process and the workflow. Our goal was to place LTCM during the same encounter once an outpatient stroke or neurology provider prescribed it. We also aimed at placing LTCM on the same day or next business day post-discharge when an LTCM was indicated but not applied in the inpatient setting. No appointment was required for device placement. The project was approved by the Geisinger Institutional Review of Board.

### Patient Selection and Enrollment

We prospectively followed every patient who had a request from one of our inpatient or outpatient stroke or neurology providers to receive an LTCM from December 2016 to 2018. We included patients older than 18 years old who had a diagnosis of cryptogenic stroke (according to the TOAST criteria) (11), TIA, or syncope with unknown etiology. Patients who were required an LTCM were enrolled and provided one at the end of the visit. For the hospitalized patients, when an LTCM was indicated but was not applied, a request was sent to the stroke clinic, and the patient received an LTCM at the stroke clinic upon discharge. Patients often received the device on the day of discharge. If a patient was discharged on weekends or after business hours, the patient received the device on the next business day. Every stroke patient included in this study had a neuroimaging consistent with the diagnosis of an ischemic stroke. Patients with TIA also had a brain MRI or a head CT scan. Every post-hospital discharged patient had routine ECG, cerebral vessel imaging, inpatient telemetry recording, transthoracic or transesophageal echocardiography, and patent foramen ovale screening when indicated. There were no exclusion criteria. Patients who did not receive the device due to insurance denial, lack of coverage, lack of consent, or other barriers such as the device not being able to detect heart rate or device detachment before the study completion, were also included among the total number of patients in the study.

All the LTCMs were placed in our two outpatient hospital-based stroke clinics. The stroke clinics at our institution are primarily staffed by stroke neurologists or stroke advanced practitioners. Patients received relevant education, written instructions, and a prepaid postage box prior to the device placement. After completion of telemetry, patients were instructed to detach the device and mail it out to the device manufacturer. We confirmed that each device was recording properly before discharging the patient from the clinic. The patient had the option of calling the clinic with any questions. We partnered with our cardiology team who agreed to verify the initial findings as reported by the device manufacturer certified cardiographic technicians. The cardiology team also agreed to facilitate rapid evaluation of patients who required urgent cardiac evaluation and intervention (e.g., electrocardiographic pauses). We received an email notification as soon as the study report was available. The initial report was also faxed to our clinic. If the patient had any of the critical findings defined as MD Notification Criteria (Table 1), the on-call stroke neurologist was notified by phone.

**Table 1:** MD notification criteria.

**Notification criteria**
Wide QRS Tachycardia >120 bpm^*^ (sustained for >30 s) (Includes monomorphic VT^a^, polymorphic VT, VF^b^)
Complete Heart Block
Symptomatic 2nd Degree AV^c^ Block, Mobitz II
Pause >6
Symptomatic Bradycardia <40 bpm (sustained for >30 s)
Atrial Fibrillation/Atrial FlutterAverage Heart Rate <40 bpm or >180 bpm (sustained for 60 s)
Narrow QRS Tachycardia >180 bpm (sustained for 60 s)

a Ventricular Tachycardia.

b Ventricular Fibrillation.

c AtrioVentricular.

* *Beats per minute*.

For the purpose of this study, we recorded patients' demographics and the parameters as described above. The feasibility measures included LTCM order to placement, wear time, analyzable time, as well as patients' out of pocket cost. Out-of-pocket costs refer to the portion of a medical expenses that a patient expects to pay for a medical diagnostic or treatment. The preliminary efficacy measures included the diagnostic rate of PAF. We compared our results to the national data (unpublished report) provided by the device manufacturer.

### Statistical Analysis

We summarized all continuous variables as mean ± SD (normal distribution) and as median with IQR (skewed distribution). We summarized all categorical variables as percentages with their corresponding 95% CIs. We used SPSS 24.0 (Chicago, Ill., USA) for our statistical analysis.

## Results

**Figure 1:**
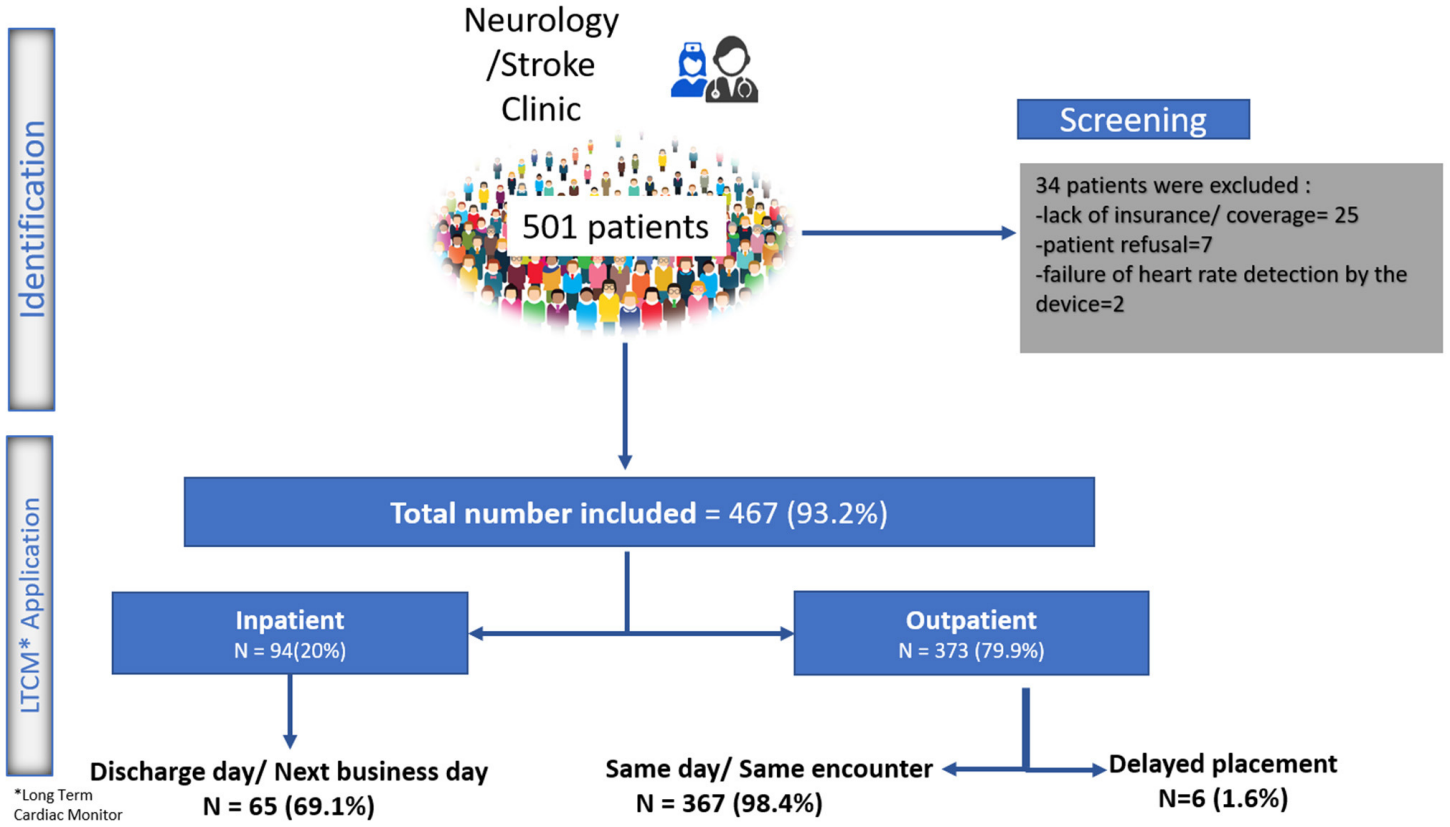
Study flow diagram.

**Figure 2:**
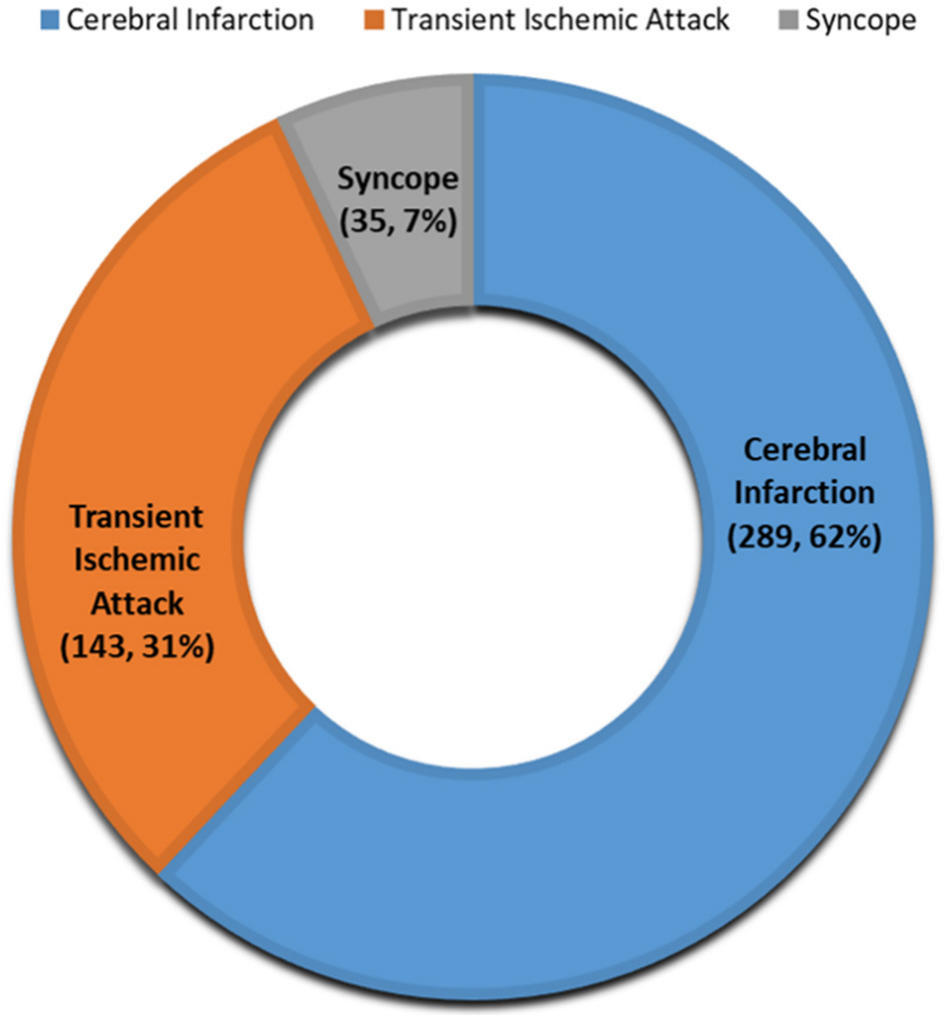
Indications for long term cardiac monitoring.

From December 2016 to 2018, 501 patients received a prescription for LTCM from one of our inpatient or outpatient stroke or neurology providers. Twenty-five patients were not able to receive the device due to various insurance-related barriers, and seven patients refused the device due to out-of-pocket cost. In two patients, the device could not detect the heart rate even after three attempts. A total of 467 (93.2%) patients (mean age 65.9 ± 13 years old; men: 48%) received LTCM. A total of 373 (79.9%) patients received the LTCM prescription in our outpatient stroke clinic. The remaining patients had a prescription from our inpatient stroke providers and received their LTCM post-hospital discharge in the stroke clinic (Figure 1). The majority of patients (92.5%) had a diagnosis of stroke or TIA (Figure 2).

### Feasibility Measures

All the 373 outpatient requests for LTCM, except six, were processed and the patch was placed during the same outpatient encounter and before the patient left the clinic. Out of 94 inpatient requests, 69.1% were fulfilled on the same day or next day (within 24 h) following discharge. The median and mean time interval between index stroke to LTCM were 4 and 4.6 days, respectively. Reasons for delay in placing LTCM were: Need for additional insurance information; Pending approval; Patient's uncertainty; Weekend discharge; Lack of supply (Figure 1).

The mean and median wearing time for LTCM based on monitoring time and recording data were 12.1 and 13.8 days, respectively. We had a total of 16 patients whose patch was detached in the first few days. Eleven patients returned to the clinic and received a new patch; Five patients refused to return and sent the device back for interpretation. The average wearing time in our population was higher compared to the national data provided by the device manufacturer (12.1 vs. 9.9 days). The average analyzable time among our patients was 95.0% (Table 2). The mean time from LTCM application to the preliminary findings was 21.4 ± 2.5 days. All the administered devices were returned for interpretation. We were able to deliver the results to all the patients by phone or during follow up visits. None of the patients missed the follow-up visits for further clinical evaluation when it was indicated. Two patients died due to stroke complications before the results were interpreted.

**Table 2:** Patients' demographics and clinical information.

**Patient profile**	**Geisinger** ***n*: 467**	**Device manufacturer**
Mean age (years)	65.9	60.2
Gender (Male)	52.8%	–
History of high blood pressure	70.8%	–
History of diabetes	20.1%	–
Median initial NIH stroke scale, interquartile range	7(4-13)	–
**FEASIBILITY MEASURES**		
Mean wear time (days)	12.1	9.9
Median wear time (days)	13.8	12.3
Mean analyzable time	95.0%	95.9%
Median analyzable time	99.1%	99.1%
Average days to 1st symptomatic arrhythmia (days)	4.1	3.7
Maximum days to 1st symptomatic arrhythmia (days)	12.2	14.0
Median days to 1st symptomatic arrhythmias (days)	3.1	2.4
Out of pocket >$100 (%)	16%	–
**PRELIMINARY EFFICACY MEASURES**		
Arrhythmias	384 (82.2%)	72.6%
Any arrhythmias (excluding continues atrial fibrillation)	383 (82.0%)	70.0%
Multiple arrhythmias (≥2)	122 (26.1%)	25.4%
Patients reporting symptomatic events	306 (65.5%)	73.7%
Patients reporting symptomatic events correlated with a detected arrhythmia	22 (4.7%)	16.9%
Any arrhythmias meeting “MD notification criteria”	21 (4.5%)	5.3%
Ventricular tachycardia (≥4 beats)	119 (25.5%)	22.6%
Ventricular tachycardia (≥8 beats)	51 (10.9%)	8.7%
Pause (>3 s)	10 (2.1%)	4.0%
Atrioventricular block (2nd degree Mobitz II or 3rd degree)	11 (2.4%)	1.6%
Supraventricular tachycardia (≥4 beats)	360 (77.1%)	60.9%
Supraventricular tachycardia (≥ 8 beats)	273 (58.5%)	46.3%
Supraventricular tachycardia (≥30 s)	18 (3.9%)	5.8%
All atrial fibrillation and atrial flutter	18 (3.9%)	13.0%
Continuous atrial fibrillation and atrial flutter	1.1%	5.5%
Paroxysmal atrial fibrillation and atrial flutter	2.8%	7.5%
Paroxysmal ventricular tachycardia	0.0%	0.0%

### Preliminary Efficacy Measures

Out of 467 patients, 384 patients (82.2%) had some arrhythmias during the 2-week monitoring period. Although 306 (65.5%) patients manually activated the monitor after experiencing some symptoms, only 22 patients (4.7%) activated the monitor during an actual cardiac event that was correlated with a recorded arrhythmia. Out of 467 patients, 18 (3.9%, 95%CI: 2.4–6.0) had at least one episode of PAF that was sustained for more than 30 s. The rate of continuous atrial fibrillation was 1.1%, and the rate of PAF was 2.8%. The rate of PAF was 5.9% (95%CI: 3.5–9.2) among patients with the diagnosis of stroke. Twenty-two (4.7%, 95%CI: 3.0–7.0) patients had some findings that met the MD notification criteria (Table 1). A total of 122 (26.1%) had multiple (≥2) types of arrhythmias. We found 51 (13.3%) patients who had ventricular tachycardia with ≥8 beats. A pause of 3 s or more was recorded in 10 (2.6%) patients, whereas atrioventricular block (2nd-degree Mobitz II or 3rd Degree) was seen in 11 (2.9%) patients (Table 2). Supraventricular tachycardia (SVT) of more than 4 beats was present in up to 77.1% of patients. SVT of eight beats or more was present in 58.5% of patients. The mean, maximum, and median number of days to the 1st symptomatic arrhythmia among our cohort was 4.1, 12.2, and 3.1 days, respectively.

### Cost

**Figure 3:**
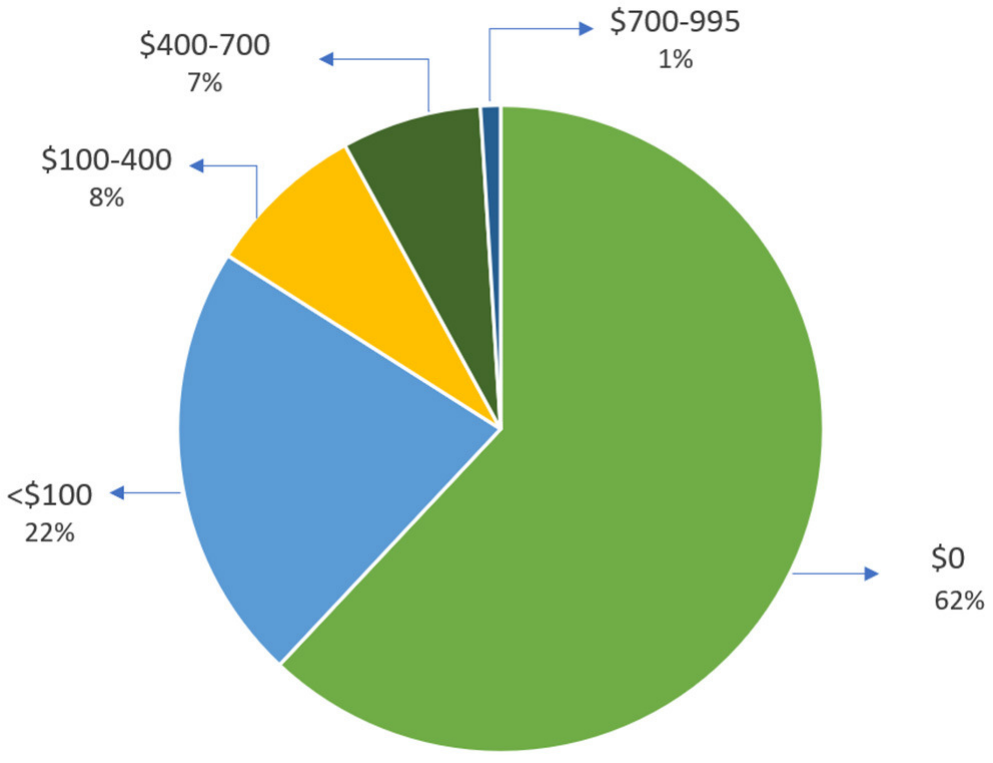
Patient out of pocket cost comparison.

Out of 467 patients, 392 (84%) patients had a total cost of <$100 for this device. Eight percent of patients had a total cost between $100–$400, and only one percent were charged between $700–$995 (Figure 3).

## Discussion

The results of our study indicate that it is feasible to implement a fast-track program in a stroke clinic with proper training of neurology providers, clinic support staff, and cardiology support. Over 93% of patients who had the request for LTCM placement received the heart monitoring device. More than 98% of patients who presented to the outpatient stroke clinic received the monitor during the same visit. Our average wearing time was 12.1 days out of 14.0 days, which was comparable to the data provided by the device manufacturer (9.9 days). Although we cannot explain all the factors contributing to our well-performing wearing time; This could probably be due to the seriousness of the primary diagnosis (stroke) and related co-morbidities that played an essential role in patient compliance and contributed to a longer wearing time compared to the national average data provided by the device manufacturer. The education provided by the clinic staff might also play a role.

To the best of our knowledge, this is the first study that evaluated the feasibility of a fast-track LTCM program in a stroke clinic. Limited evidence has shown that the self-application of this type of LTCM might also be feasible. A recently published study (12) compared the analyzable time between patients who were taught over the phone to apply the patch and a control group. The study found no significant difference in analyzable time. However, the study had a small sample size of 30 patients. Another study which was conducted in Germany investigated whether implantation of an insertable cardiac monitor during an outpatient follow-up is feasible among patients with cryptogenic stroke. The study had a similar conclusion regarding the feasibility of insertable cardiac monitor in an outpatient clinic to detect atrial fibrillation (13).

Several studies have indicated that prompt initiation of heart monitoring is critical for timely diagnosis of AF (13, 14). It has been shown that the effective diagnosis of atrial fibrillation decreases the risk of recurrent stroke (14–16), and death (17, 18) and would improve patients' outcomes (19–21). While we did not measure patient satisfaction in our study, evidence suggests that same-day scheduling and diagnostic testing reduce waiting time (22), unnecessary referrals and costs (23, 24), and perhaps engage physicians as well as improve patient satisfaction (25, 26). This is important for stroke patients, as they often have some degree of disability and limited mobility. Monitoring for arrhythmias should be considered to be a standard practice in a stroke unit or clinic, as it helps to detect underlying PAF, determine stroke etiology, and provide optimized and timely secondary prevention (27).

### Detection Rate of Atrial Fibrillation and Flutter

The current evidence suggests that extended heart monitoring beyond 24–72 h could increase the frequency of PAF detection anywhere from 1.0 to 6.0% (28). The rate of PAF detection in our study (3.9%) is lower than the national data provided by the device manufacturer (13%). This is possible as our sample consisted of a heterogenous stroke and TIA population. Since there is no biomarker for TIA, it is possible that many of the TIA patients in this study had other etiologies for their transient neurological symptoms. The rate of AF detection could also be highly variable depending on the patient pool. Different studies have reported a wide range of AF following stroke and TIA (29).

Underlying PAF was seen among 5.9% of our confirmed stroke patients. The rate of PAF detection reported by the device manufacturer based on their patient pool was 7.5%. Several studies have shown that the rate of PAF in patients with cryptogenic stroke can be variable and is mainly dependent on patients' age and history of ischemic heart disease (30, 31). A published review of studies (32) that used similar LTCM device indicated an atrial fibrillation detection rates ranged from 3.5 to 58.1% for prescribed recording durations up to 14 days with a mean pooled rate of 12.7%. Two other systematic reviews of monitoring durations ≥7 days following an ischemic stroke or TIA have reported PAF detection rates of 6% and 15% (33, 34). Variation in study designs, inclusion/exclusion criteria, and monitoring duration may account for the wide range of PAF detection among patients with stroke.

### Detection Rate of Other Arrhythmias

The study results showed a high rate (77.1%) of supraventricular tachycardia (SVT) detection among the patient population. Although this rate may seem higher as compared to the pooled data from 22 other studies (60.9%) (32), the results of a comparable study (35) indicate that patients with stroke and TIA may have a higher rate of SVT as compared to patients with no history of cerebral ischemia. A recent study of patients with pacemakers or implantable cardioverter-defibrillators showed subclinical atrial tachyarrhythmia preceding the development of clinical AF (36). Subclinical atrial tachyarrhythmia is a form of SVT, and is defined as an episode of rapid atrial rate (≥190 beats per minute) lasting more than 6 min. Healey and colleagues (36) also showed a nearly 2.5-fold increased risk of stroke among patients with atrial tachyarrhythmias, regardless of the presence of AF. Nevertheless, the high burden of SVT can be an indicator of PAF and it might be reasonable to consider implantable loop recorder for further monitoring among some of these patients.

More than 80% of patients had some arrhythmias during the 2-week course of the heart monitoring process. This rate was slightly higher when compared to the national rate provided by the device manufacturer (70%) (Table 2). One reason for the higher rate could be the older age in our cohort. Other studies among stroke patients have also suggested that large strokes and the affected hemisphere are associated with a higher risk of arrhythmias (37–40). There is also a high frequency of arrhythmias in the first few days after a stroke (37).

Our results indicate that there were no regularly corresponding arrhythmias when the LTCM was manually triggered by patients. A similar study found that more than 53% of patients who experienced symptoms and marked the timing of the event on LTCM, did not have a corresponding arrhythmia (41). One reason for the non-detection of arrhythmia could be that the patients triggered the heart monitor late and therefore the event was missed (42), or patients with AF did not feel any palpitations. In the European Heart Survey on Atrial Fibrillation, more than half of patients (54%) were asymptomatic at the time of the survey, and the lowest symptom burden was reported in PAF (43). These observations suggest the ability of long-term monitoring to rule out cardiac arrhythmias as the origin of patients' symptoms.

Over 80% of patients had a <$100 out-of-pocket cost for this device. The data suggests that our approach might be more cost-effective than conventional heart monitoring using Holter monitor which has a low sensitivity in detecting PAF in stroke (10, 44). Although continuous ECG recording of up to 48 h with multi-lead Holter monitors is commonly utilized as an initial option for screening and detection of PAF among stroke patients, multiple studies have demonstrated a higher detection rate with longer monitoring time (10, 45). Despite promising results from implantable cardiac monitors in long-term PAF detection, the invasive nature of these monitoring techniques and cost may prevent their widespread use and they may not be a feasible first-line cardiac monitoring option for some of the stroke patients.

Our study was a prospective study with a large sample size; however, the study had some limitations. We did not record the patients' long-term findings, and outcomes (late diagnosis of PAF, recurrent stroke, etc.). The evaluation of feasibility was the focus of this study. The time from stroke to monitoring and functional status at initiation of monitoring were also not recorded. We were not able to measure the inter-rater agreement between the manufacturer algorithm, certified cardiology technicians, and our cardiologists. We did not measure patient satisfaction. While we compared some of our findings to the national data provided by the device manufacturer, we did not have a comparison or control group within our institution. Although our staff and providers embraced the work-flow and this initiative is now part of our stroke clinic, we did not systematically measure their satisfaction and engagement. Optimal evaluation of feasibility may necessitate exploring these parameters. Implementation in other institutions and populations is necessary to evaluate for generalizability.

## Conclusion

Our findings demonstrate that it is feasible to implement a fast-track long term continuous heart monitoring as part of a stroke clinic with proper training of stroke providers, clinic staff, and support from a cardiology team.

## Data Availability Statement

All datasets generated for this study are included in the article/supplementary material.

## Ethics Statement

The studies involving human participants were reviewed and approved by Geisinger Institutional Review Board. Written informed consent for participation was not required for this study in accordance with the national legislation and the institutional requirements.

## Author Contributions

RZ, AK, and VA contributed equally to manuscript conception, design, revisions, and approved the submitted version. FI, AS, MA, MM, and NH authors contributed to manuscript revision, read, and approved the submitted version.

### Conflict of Interest

The authors declare that the research was conducted in the absence of any commercial or financial relationships that could be construed as a potential conflict of interest.
